# Deep Transfer Learning-Based Approach for Glucose Transporter-1 (GLUT1) Expression Assessment

**DOI:** 10.1007/s10278-023-00859-0

**Published:** 2023-09-05

**Authors:** Maisun Mohamed Al Zorgani, Hassan Ugail, Klaus Pors, Abdullahi Magaji Dauda

**Affiliations:** 1 Faculty of Engineering and Informatics, School of Media, Design and Technology, University of Bradford, Richmond Road, Bradford, BD7 1DP UK; 2 Institute of Cancer Therapeutics, University of Bradford, Richmond Road, Bradford, BD7 1DP UK

**Keywords:** GLUT-1 scoring, Deep transfer learning, Colorectal cancer, IHC image analysis, Tumour hypoxia

## Abstract

Glucose transporter-1 (GLUT-1) expression level is a biomarker of tumour hypoxia condition in immunohistochemistry (IHC)-stained images. Thus, the GLUT-1 scoring is a routine procedure currently employed for predicting tumour hypoxia markers in clinical practice. However, visual assessment of GLUT-1 scores is subjective and consequently prone to inter-pathologist variability. Therefore, this study proposes an automated method for assessing GLUT-1 scores in IHC colorectal carcinoma images. For this purpose, we leverage deep transfer learning methodologies for evaluating the performance of six different pre-trained convolutional neural network (CNN) architectures: AlexNet, VGG16, GoogleNet, ResNet50, DenseNet-201 and ShuffleNet. The target CNNs are fine-tuned as classifiers or adapted as feature extractors with support vector machine (SVM) to classify GLUT-1 scores in IHC images. Our experimental results show that the winning model is the trained SVM classifier on the extracted deep features fusion *Feat-Concat* from DenseNet201, ResNet50 and GoogLeNet extractors. It yields the highest prediction accuracy of 98.86%, thus outperforming the other classifiers on our dataset. We also conclude, from comparing the methodologies, that the off-the-shelf feature extraction is better than the fine-tuning model in terms of time and resources required for training.

## Introduction

It is known that most solid tumour tissues have an abnormal vasculature resulting in poor delivery of oxygen to localised regions. Therefore, these regions are characterised by low oxygen concentration, known as tumour hypoxia [1]. Hypoxia is currently receiving attention as the centre for the hallmarks of cancer; this is because of its major characteristics of chemotherapy and radiotherapy resistance and a major prognostic factor [2]. Oxygen gradient in the order of diffusion and consumption plays a critical role in the dynamics of the tumour microenvironment, and this creates room for the development of hypoxic regions in almost all solid tumours [3]. The hypoxic regions in all solid tumours vary in size and extent due to the different gradients and supply as well as distribution of oxygen in these regions [4]. The need for oxygen in solid tumours differs; some tumours that are irregular and disorganised require a reduced oxygen gradient, whilst others require an increase in oxygen demand which arises as a result of tumour metabolism [5]. The imbalance between oxygen demand and supply in solid tumours makes hypoxia a unique hallmark of cancer as it creates room for tumours to more adverse features that include epithelial–mesenchymal transition (EMT) and mesenchymal–epithelial transition that produces cancer stem cell (CSC) niche, resistance to chemotherapy and radiotherapy resistance, poor clinical prognosis and immune damping as well as increase genomic instability and increases cells abilities to evade apoptosis [4, 6]. Hypoxia and angiogenesis work hand in hand for all solid tumours to be fully established and therefore are linked to aggressiveness and metastasis of solid tumours, hence among the most relevant micro-environmental factors [5]. Tumour hypoxia leads to the production and activation of hypoxia-inducible factor (HIF) family members, including HIF-1 and HIF-2; these HIFs’ proteins later activate more genes that promote hypoxia such as the GLUT-1 and carbonic anhydrase-9 (CAIX) [7] which are both well-known membrane-bound biomarkers that induce and increase the hypoxic condition in solid [8]. Thus, the GLUT-1 as a cell surface receptor is one of the genes associated with tumour hypoxia in different forms of cancer including breast, prostate and colorectal and hence classed as a hypoxic stem cell marker cancer [9]. The semi-quantitative scoring system is employed for estimating the amount of GLUT-1 expression. Several studies [9–12] have employed the semi-quantitative scoring system to assess the level of hypoxia marker using the GLUT-1 protein expression. The clinical significance of the different scores of tumour hypoxia therapy is aimed at understanding how much hypoxia is developed and spread across solid tumours and cells. The deeper the hypoxic environment, the more difficult it gets for chemotherapy and radiotherapy to reach tumour sites. Hence, there is a need for the use of hypoxia-activated pro-drugs (HAPs) as targeted therapy. For the apparent progress in cancer therapies, the visual examination still is the standard way of measuring tumour hypoxia markers in clinical practice, which is affected by inter-observer variation [13–15]. In addition, commercial software such as QuPath imaging software requires an expert pathologist to quantify the intensity of GLUT1 ImmunoStaining in tumour cells. Therefore, one of the steps of tumour therapy development would be promoted by a robust automated scoring method to facilitate clinical tests, improve the diagnosis quality, avoid the variation among histopathologists and reduce the time and costs of diagnoses. This, in turn, results in better assessment outcomes and improved patient experience [15].

Traditional machine learning-based methods rely heavily on extracting some specific visual features manually from images. So, such hand-crafted features can only deal with some low-level information about images. Furthermore, the relevant domain knowledge is necessary to select useful features, which can be greatly muddled by subjective extractor bias. In contrast, deep learning-based methods can extract high-level abstract features from images automatically in a standardised way. CNNs are one form of deep learning that can extract the image’s hierarchical features at multiple layers, i.e. the features at each layer are computed from the representations of the previous layer. Such hierarchical features can be learned gradually from low-level to high-level through a deep architecture [15]. Thus, multi-level abstraction enables deep learning networks to be well suited for discovering the complex structures within high-dimensional data, such as whole slide images [16]. In addition, such CNNs have the ability to learn complex mapping functions directly from input data, without the help of human-crafted features [16].

Although deep learning algorithms achieved state-of-the-art results in the digital histopathology domain, they have some unique challenges in their implementation. One of the major challenges is the large number of annotated images needed for training deep CNNs that may not be available, especially in the digital histopathology domain [16]. In contrast, training deep CNNs with limited training data leads to over-fitting and a poor generation of features on data. Over-fitting is critical when the data contain high image appearance variance, which is usually common in digital histopathological images. Furthermore, training deep CNN from scratch requires high computational costs and extensive memory resources and time. Thus, such approaches have practical limitations in the digital histopathology field [14]. In recent years, there has been a debate that the methodology of deep transfer learning could tackle the aforementioned challenges more effectively. Deep transfer learning is leveraged as a helpful tool to overcome the data scarcity problem in the digital histopathology field. Therefore, this work has adopted the most common deep transfer learning to present an automated method for scoring the tumour hypoxia marker using the GLUT-1 protein expression. Towards the end, this work proposes alternative tumour hypoxia scoring evaluation using deep learning technology to objectively predict those patients that require targeted therapy HAPs instead of chemotherapy or radiotherapy.

The rest of the paper is organised as follows: the related works are briefly discussed in the second section. The proposed methodology is explained in the third section. The carried out experiments and their results are presented in the fourth section. Lastly, the fifth section concludes the paper.

## Related Work

The traditional IHC scoring methods rely on hand-craft features. This is due to the lack of IHC images in clinical practice. So IHC images need the high cost of antibodies, autostainer machine equipment and the complex laboratory process. To our knowledge, no work has been done for the automatic GLUT1 scoring using deep learning techniques. Therefore, this section reviews some of the works that are similar to our work. These works have participated in the contest human epidermal growth factor receptor 2 (HER2) scoring challenge on invasive breast cancer images. It was organised by the University of Warwick, the University of Nottingham and the Academic–Industrial Collaboration for Digital Pathology. It aimed to advance automated methods for HER2 scoring on IHC-stained images. HER2 expression is used as a predictor of invasive breast cancer progression in clinical practice. More information on the HER2 Challenge dataset can be found in the paper published by Qaiser et al. [17].

Since the HER2 scoring challenge contest, several studies [18–21] have been carried out by using traditional machine learning methods for HER2 scoring. Cordeiro et al. [18] have utilised and compared the SVM, K-nearest neighbours (KNN), multi-layer perceptron classifier (MLP) and decision tree classifiers for HER2 scoring according to image patch level and patient level with colour and texture features. Mukundan [19] has employed uniform local binary pattern (ULBP), characteristic curves, entropy and energy features with logistic regression and SVM classifier to score HER2-stained tissue samples. Tewary et al. [20] have utilised colour space-based membrane extraction followed by an SVM classifier for HER2 scoring. Chang et al. [21] have employed the colour channel to extract the morphology, texture and intensity features, and then they were utilised for training the SVM classifier. But these methods depend on extracting visual features manually from images which in turn requires an expert pathologist.

The others [17, 22–25] investigated deep learning methods. This is due to deep learning methods providing impressive results in many applications of histopathological image analysis. Starting from [17], Qaiser et al. have presented the winning teams of the HER2 challenge contest that employed CNN-based methods. The winning teams leveraged deep transfer learning methodologies to address the lack of annotated training images in the HER2 challenge dataset. They have fine-tuned the latest pre-trained CNNs at the time as classifiers for HER2 scoring. For instance, the MUCS team [17] submitted three versions, the AlexNet model was adapted to MUCS-1 and MUCS-2, and the GoogLeNet model was adapted to MUCS-3. MUCS-1 network is fine-tuned to classify four output classes that correspond to HER2 scores from 0 to 3 +. MUCS-2 and MUCS-3 had an additional output class for the background. The background class contained the regions with texture having only a weak appearance of nuclei (without blueish or brownish colour). MTB NLP team [17] trained the modified architectures of AlexNet and VGG-16 and then employed a random forest classifier to produce the final class probabilities for each score. In a similar manner, the VISILAB team [17] fine-tuned the GoogLeNet model, and the FSUJena team [17] fine-tuned the AlexNet model to four HER2 scores. Whilst in [22], the authors have used direct feeding to the AlexNet model for HER2 scoring. In [23], the authors have proposed Her2Net architecture with LSTM recurrent network for segmenting and labelling the HER2-stained tissue samples. In [24], the authors have applied super-pixel-based tissue region segmentation to extract colour and texture features and then followed by SVM to distinguish epithelial and stromal regions, which are scored using a modified UNet model. In [25], the authors utilised deep transfer learning with fine-tuned five pre-trained CNNs by fully connected dense layers for 3 classes and then presented a collective voting scheme for HER2 scoring. They have adopted VGG16, VGG19, ResNet50, MobileNetV2 and NASNet-Mobile architectures for image-based and patch-based labelling.

In this work, we have explored the common deep transfer learning methodologies to score GLUT-1 protein expression in a colorectal cancer tissue microarray (TMA) of IHC images. We have investigated and compared the predictive performance of six different pre-trained CNNs architectures: AlexNet, VGG16, GoogLnet, ResNet50, DenseNet-201 and ShuffleNet. These architectures were fine-tuned as classifiers or adapted as extractors to get the transferable off-the-shelf features on our IHC image dataset.

## Materials and Methods

In this section, we first present the acquisition approach of dataset images and then outline the essential steps to be followed in dataset image preparation. Next, we show the appropriate choice of architecture and methodology for our application. Finally, the proposed methodology for the discrimination of the GLUT1 scoring is quantitatively explained.

### Image Dataset Acquisition

**Fig. 1:**
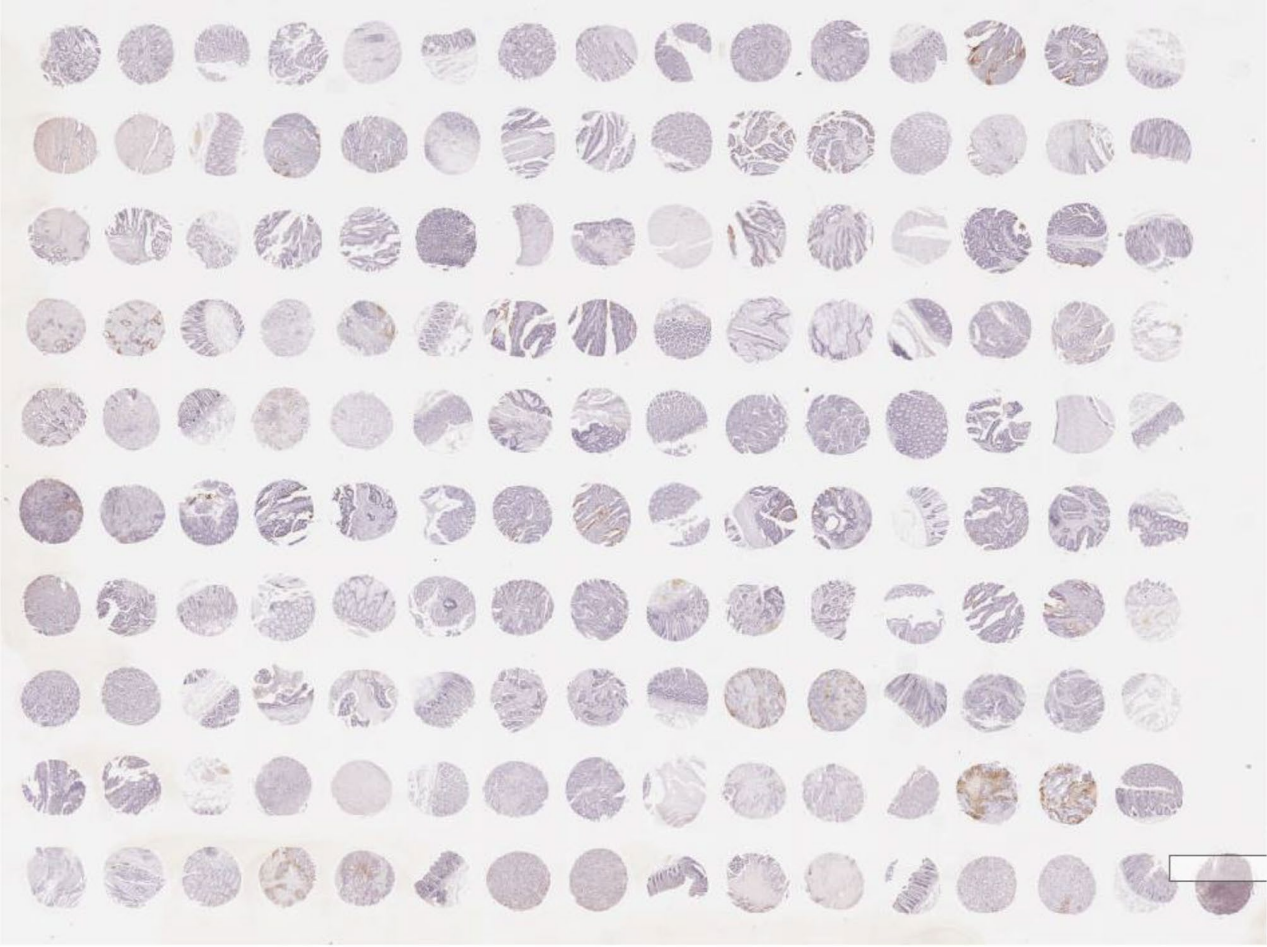
The G063 clinical sample of 150 tumour cores of Aperio Leica software

**Fig. 2:**
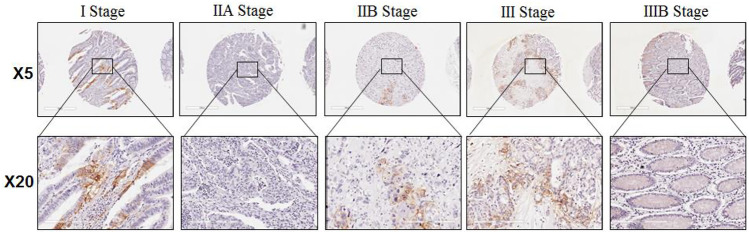
Examples of GLUT-1 expression at different stages of IHC colon adenocarcinoma images; magnification of the upper panel is × 5 (0.1 um/pixel), and the lower panel is × 20 (0.5 um/pixel)

The images were obtained from the institute of cancer therapeutics, the University of Bradford, and the necessary ethical approval has been obtained. The images were prepared by immunohistochemistry (IHC) staining for ALDH7A1 and GLUT-1 on HT-29 tissue microarray (TMA) of human clinical specimens of colorectal cancer adenocarcinoma. The IHC is a widely used technique in pathology. It is now used in all aspects of modern research to identify specific antigens within a tissue section from formalin-fixed paraffin-embedded (FFPE) tissue, e.g. in tissue microarrays (TMAs) and 3D dimensional spheroids grown from cells. The method utilises an antigen-specific antibody interaction and detection using a light microscope [26]. The TMA clinical sample slide number G063 (Biomax.us) carries 150 cores of clinical sample on the whole side in which 100 are colorectal cancer (CRC) tissues, and 50 were either malignant, adjacent tissue to the cancer tissue or normal tissues; these give a total number of 50 cases of colorectal cancer in each whole TMA slide. A whole slide IHC analysis was done to analyse ALDH7A1 protein expression in CRC clinical tissues. The clinical samples were collected between July and August 2019 from thirty-three male patients and thirteen female patients. So, these clinical samples whole slide comprises both male and female colorectal cancer patients and different age distributions from the highest age distribution of 82-year-old male patient sample with grade 1 and stage IIB CRC, whilst the least clinical sample on the whole slide was from a 33-year-old female patient sample with grade 1 and staged IIB CRC (CO1505). The IHC images were scanned using Aperio Digital Pathology Slide Scanners (Aperio AT2) and then captured at 0.5 um/pixel and 200 μm diameter. The whole cores and examples of GLUT-1 expression of IHC colon adenocarcinoma images were shown in Figs. 1 and 2, respectively.

### Annotation of Dataset Images

Two trained histopathologists scored the TMA cores according to the proportion of Glut-1 and ALDH7A1 staining in the entire cores. They have quantified the different scores using QuPath Imaging Software with version 0.2.6. Such software provides percentage (%) mean expression of protein measured as intensity per pixel. So they have analysed all the semi-quantitative scores per expression of GLUT-1 and ALDH7A1 at different stages of CRC TMA using the QuPath Imaging Software version 0.2.6. The TMA cores were semi-quantitatively scored: score 0 (no staining with 0%), score 1 (light staining with 0–5%), score 2 (medium staining with 5–15%), score-3 (heavy staining with15–30%) and score 4 (intense staining with > 30%) and a score given for whole section. The settings used were TMA de-arrayer (optional) at TMA core diameter of 1.2 mm, intensity parameters threshold of 0.1 and Max background intensity of 2. The intensity threshold parameters are under score compartment; cytoplasm, DAB OD mean, threshold 1 + 0.2, threshold 1 + 0.4 and threshold 1 + 0.6. And all of these were ran at single threshold. The examples of our dataset images are shown in Fig. 2. 

### Derivation of Dataset Images

The dataset images were obtained by hand-picking the regions of interest from TMA cores that contained the most representative samples from each class. The regions were selected at a low resolution and mapped to the highest resolution (0.5 um/pixel) to generate the patches. We derived approximately ten to twelve images for each TMA core to balance the number of images among the classes. A total of 1750 IHC images were extracted at 0.5 um/pixel. Each class contains 350 images, where we derived dataset images with size 512 × 512 pixels and then stored them in jpeg compression format.

### Colour Normalisation

For this purpose, we utilised a colour de-convolution method described in [27] for highlighting the brown-stain areas (Diaminobenzidine, DAB) of reactive membranes in the IHC image.

### Partition of Dataset Images

The dataset images were divided randomly into 80% training set (1400 images) and 20% testing set (350 images); i.e. each class was trained with 280 images and tested with 70 images. The testing set does not utilise for training our proposed architectures.

### Augmentation of Dataset Images

Several studies [16, 28] have observed that the limited amount of training data is one of the major challenges in deep learning. In this regard, data augmentation is one of the possible solutions to create additional artificial training images through some transformations for learning deep features from the images and thus increasing the deep network performance. In this study, we augmented the training images of our dataset by rotated them with angles of 90, 180 and 270 degrees and then flipped them in the horizontal and vertical direction. This is to enlarge the training images size without affecting the quality of input images [29] and avoid the problems of over-fitting and poorly generation of features [30].

### Standard Performance Evaluation Metrics

The performance of different classifiers is evaluated according to standard metrics formulated for multi-class classification as depicted in Eqs. 1, 2, 3 and 4:

- Accuracy metric: this criterion is used to measure a classifier’s ability for predicting actual classes correctly. It is formulated as
  1 $$Acc=\frac{1}{L}\sum_{i=1}^{L}\frac{t{p}_{i}+t{n}_{i}}{t{p}_{i}+t{n}_{i}+f{p}_{i}+f{n}_{i}},$$
- Recall metric: it is also known as sensitivity; this criterion is used to measure a classifier’s ability for predicting each individual class correctly. It is formulated as
  2 $${R}_{i}=\frac{t{p}_{i}}{t{p}_{i}+f{n}_{i}},$$
- Precision metric: this criterion is used to measure a classifier’s ability for predicting relevant instances for each individual class. It is formulated as
  3 $${P}_{i}=\frac{t{p}_{i}}{t{p}_{i}+f{p}_{i}},$$
- F1-score metric: this criterion is a harmonic mean of the recall and precision metrics. It is formulated as
  4 $${F}_{1} score=\frac{2\times {P}_{i}\times {R}_{i}}{{P}_{i}+{R}_{i}},$$

where tpi is the number of true positives (i.e. the correctly classification for ith class), fpi is the number of false positives (i.e. wrongly classification for ith class), fni is the number of false negatives (i.e. missed classification for ith class), tni the is the number of true negatives (i.e. correctly classification not belong to ith class), and L is the number of classes.

**Fig. 3:**
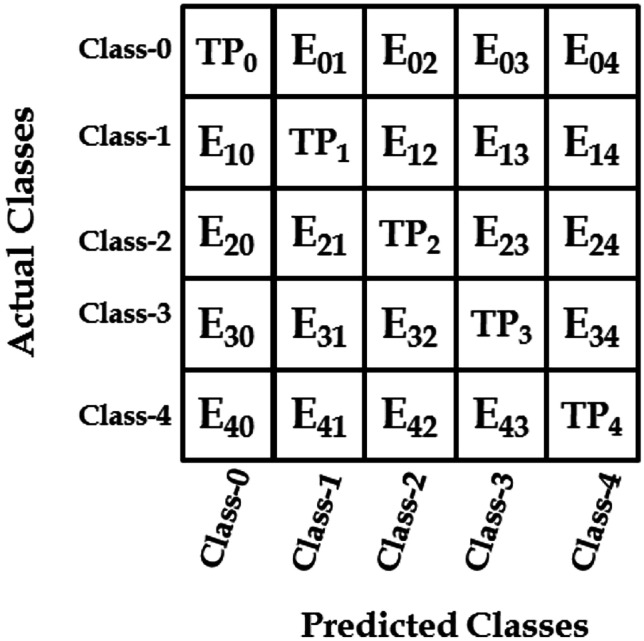
A confusion matrix for the five-class classification

In this study, standard performance metrics were extracted from the confusion matrix [31]. Thus the 5 × 5 confusion matrix for classifying five-class that utilised in this work is shown in Fig. 3. So the accuracy, recall and precision could be calculated using the following equations:

5 $$ACC=\frac{\sum_{i=0}^{L-1}t{p}_{i}}{\sum_{i=0}^{L-1}t{p}_{i}+\sum_{i=0}^{L-1}\sum_{j=0}^{L-1}{E}_{ij}}, \forall i\ne j, i=j=\left\{\mathrm{0,1},\mathrm{2,3},4\right\}$$

6 $${R}_{i}=\frac{t{p}_{i}}{t{p}_{i}+\sum_{j=0,\forall i\ne j}^{L-1}{E}_{ij}}, i=\left\{\mathrm{0,1},\mathrm{2,3},4\right\}$$

7 $${P}_{i}=\frac{t{p}_{i}}{t{p}_{i}+\sum_{j=0,\forall i\ne j}^{L-1}{E}_{ji}}, i=\left\{\mathrm{0,1},\mathrm{2,3},4\right\}$$

From Fig. 3, the term *E*_ij_ is the error classification to the actual class *i* according to predicted class *j*. By substituting into Eqs. 1, 2 and 3, we find that $t{p}_{i}+f{p}_{i}=\sum_{i=0}^{L-1}t{p}_{i}$, $f{n}_{i}=\sum_{j=0,\forall i\ne j}^{L-1}{E}_{ij}$ and $f{p}_{i}=\sum_{j=0,\forall i\ne j}^{L-1}{E}_{ji}$.

### Choice of Deep Transfer Learning Methodology

In this section, we introduce the concept of two common methodologies.

#### Fine-Tuning CNN

In this methodology, the target pre-trained CNN network layers can be replaced by new ones and then retrained on the new dataset. It is possible to fine-tune all the network layers or fix the earlier layers and only fine-tune the last layers of the network [32]. The weights in transferred layers are frozen and initialised from a source pre-trained network, whilst the weights in new layers are trained using a back-propagation algorithm [33]. In the back-propagation algorithm, the weights in the new layers are adjusted by back-propagating the new task’s errors into the transfer layers during training. It is faster than constructing a new CNN network, i.e. a pre-trained CNN on millions of images could be taken to retrain for new classification using only hundreds of images [33]. Furthermore, it gains convergence faster than learning from scratch so that it can solve the convergence problem [34].

#### Off-the-Shelf Feature Extraction

Such methodology adapts the pre-trained CNNs to extract the deep features which then are utilised to train a separate classifier for prediction without consuming time and effort for training. These extractors are characterised by the generalisation property which enables the deep features to be transferred to other applications [35]. Therefore, the generalisability property is particularly useful when there is not enough dataset for training the CNN from scratch.

#### Architecture of the Proposed Models

Choice of the appropriate architecture for a specific application is an essential step. In this work, we have investigated six pre-trained CNNs trained on the ImageNet dataset [36]:

- *AlexNet architecture* [13] was the winner of the ImageNet 2012 challenge that popularised CNNs. It contains five convolutional layers followed by rectification, three max-pooling layers and three fully connected layers.
- *VGG architecture* [37] was proposed by the Visual Geometry Group at the University of Oxford. It has a similar architecture to AlexNet with more convolutional layers. In VGG architecture, the convolutional layers use only 3 × 3 filters, as well the pooling layers employ only 2 × 2 filters. VGG16 model has thirteen convolutional layers followed by rectification, five max-pooling layers and three fully connected layers.
- *GoogLeNet architecture* [32] contains twenty-two convolutional layers with nine inception blocks and a fully connected layer. The inception module consists of four parallel convolution kernels that process the same input, and the extracted different features are then concatenated at the end. Each inception module has six convolutional layers followed by rectification.
- *Residual architecture* [38] uses skip connections to reduce the effect of the vanishing gradient problem significantly. ResNet-50 has 50 convolutional layers followed by rectification and a fully connected layer. It contains 16 residual blocks with the skip layer element-wise addition layer to enable the network to pass the features from lower to higher levels to acquire more complicated features.
- *DenseNet architecture* [39] utilises the skip connections from each layer to the succeeding layers that promote reusing the features through the entirety of the network. DenseNet-201 contains 201 convolutional layers and a fully connected layer. It has dense blocks that concatenate outputs from all the previous layers as its input.
- *ShuffleNet architecture* [40] contains grouped convolution, channel shuffling and element-wise addition of two inputs. So, it combines the characteristics of residual and dense blocks. It utilises the channel shuffle operation to overcome the consequences of using group convolutions.

**Fig. 4:**
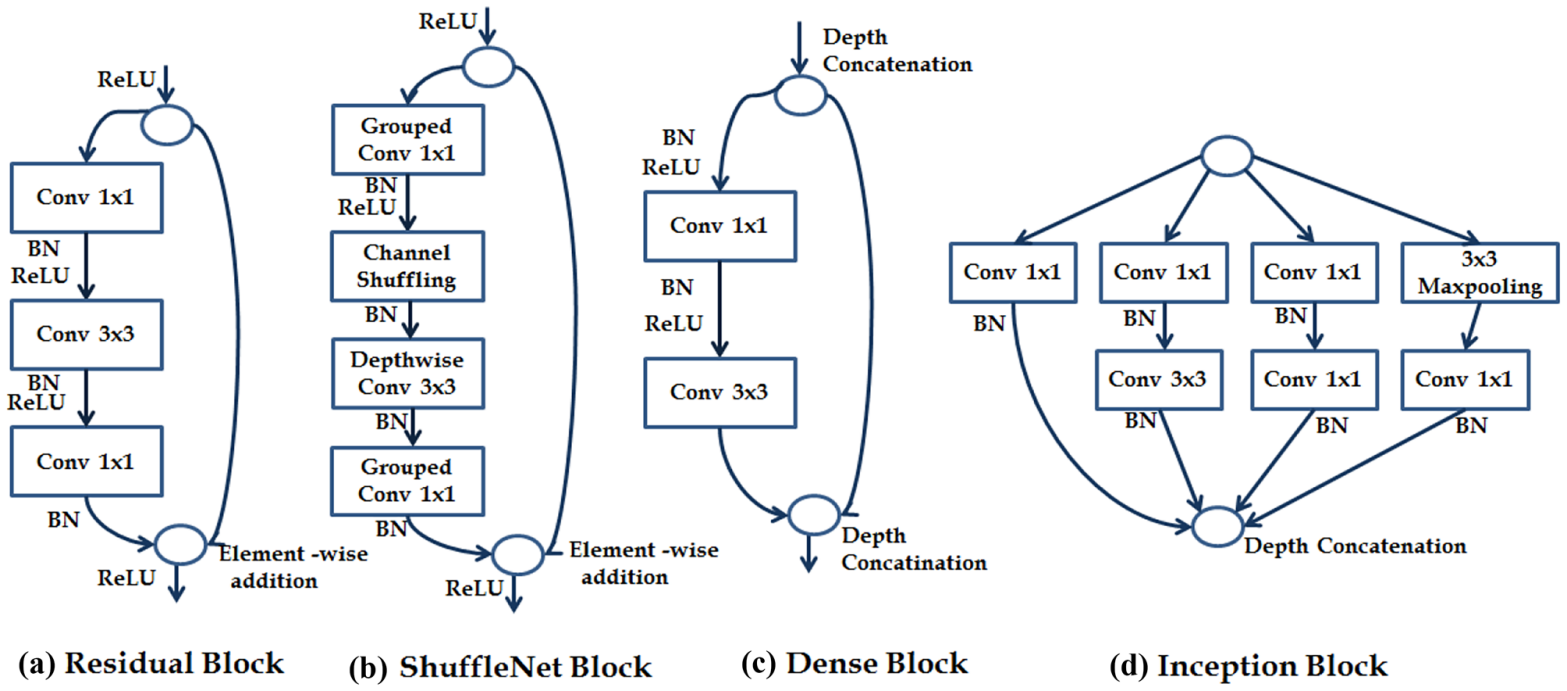
Block structure for different proposed architectures, where “Conv”, “ReLU” and “BN” represents the convolution layer, rectified linear unit and batch normalisation layer, respectively

In the AlexNet and VGG-16 models, the feature extraction part is regarded from the input layer to the last max-pooling layer, whereas the classification part is three fully connected layers. Whilst in the other models, from the input layer to the global average pooling layer is the feature extraction part, and the fully connected layer is the classification part. The different blocks for proposed architectures are shown in Fig. 4.

### Proposed Methodology

This study has investigated the common deep transfer learning methodologies: fine-tuning of pre-trained CNNs for a new classification and off-the-shelf features extraction from pre-trained models. In the first approach, we initialise the target network with pre-trained weights on the source task and then partially re-training them on the target task. Whilst the second approach extracts the deep features from the source task without re-training the network and then uses them to train a third-party classifier.

#### Fine-Tuning Target CNN Classifiers

In this experiment, we compare the performance of six different pre-trained models: AlexNet, VGG16, GoogLeNet, ResNet-50, DenseNet-201 and ShuffleNet. We carried out our experiments by fine-tuning these target models as classifiers. We fine-tuned the fully connected layer of these models by replacing the last three layers with new layers for five classes; i.e. the new layers are mapped to our dataset. We specifically replace the fully connected, softmax and classification layers with three corresponding new layers. Subsequently, the weights in transferred layers were preserved, whilst the weights in new layers were updated continuously using the back-propagation algorithm.

#### Tuning Target CNN Feature Extractors

In this experiment, we have compared the performance of SVM classifiers that were trained on the extracted deep features from a specific layer in the proposed networks as follows: global average pooling “avg-pool” layer for GoogLeNet, ResNet-50, DenseNet-201 and ShuffleNet, whilst “f7” fully connected layer for AlexNet and VGG16. The SVM classifiers were tuned and trained on the extracted deep features using the training set and then evaluated using the test set. In the sequel, the extracted deep feature vectors from fully connected layer fc7 of AlexNet and VGG-16 will be referred to as Feat-Alex and Feat-Vgg16, respectively. As the same, the extracted deep feature vectors from the global average pooling layer of GoogLeNet, ResNet-50, DenseNet and SuffleNet will be referred to as Feat-GoogLe, Feat-Res50, Feat-Dense and Feat-Suffle, respectively.

## Experiments and Results

**Fig. 5:**
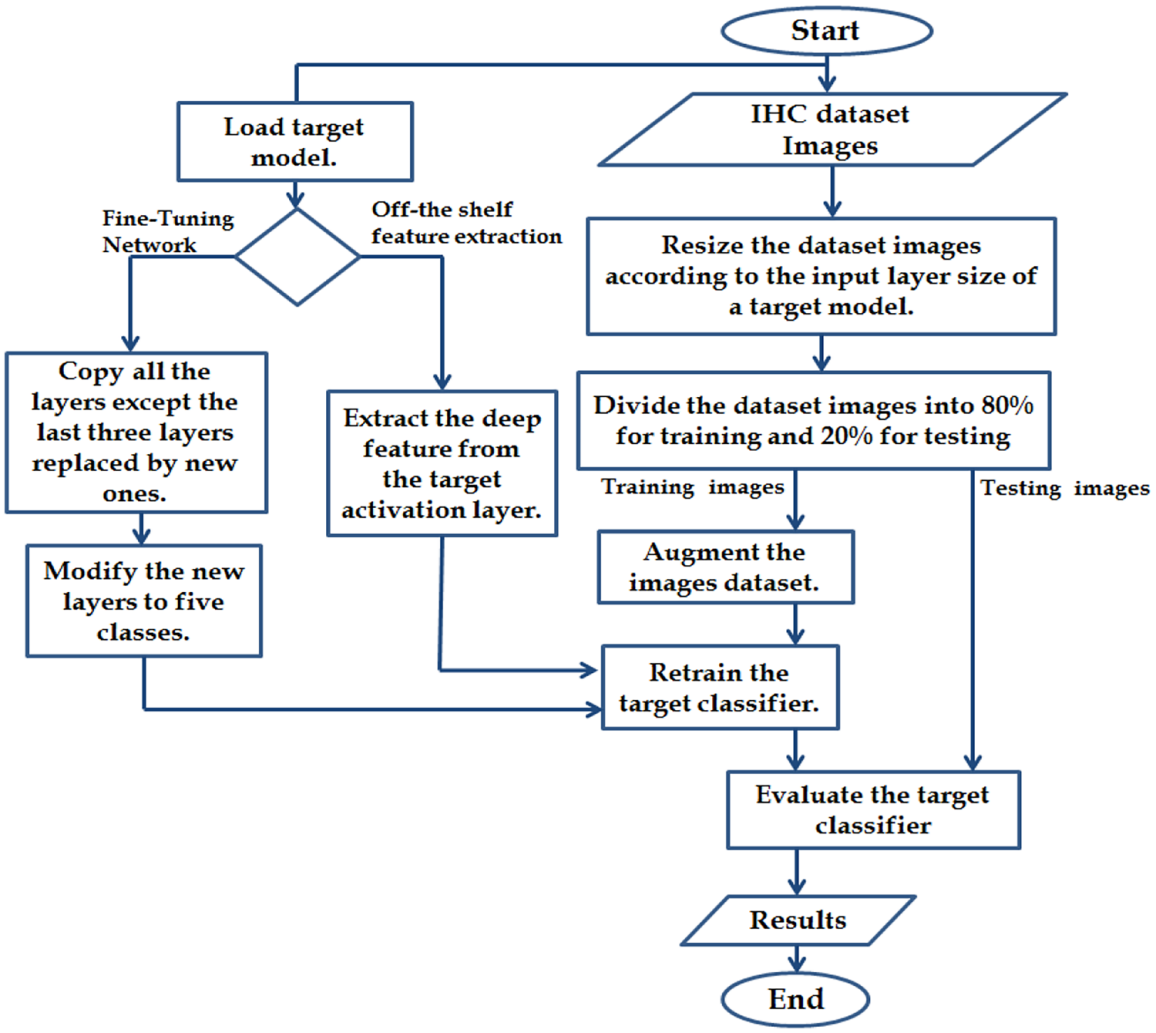
Flowchart diagram for the proposed methodologies

Three experiments are carried out using our dataset images. The experiments are implemented in MATLAB R2020a on a desktop computer with a 3.60-GHz Intel® CPU, Dual-Core-i7-7700, 32 GB RAM and NVIDIA GeForce GTX 1070 GPU. In our experiments, the target CNN models were fed with the augmented IHC images, which were resized according to their input layer size. AlexNet architecture requires input images of size 227 × 227 × 3, whilst other architectures require input images of size 224 × 224 × 3, where 3 is the number of colour channels. Now, the dataset images are ready to employ according to the used approach. In the fine-tuning classifier, augmented data is fed to the target networks for training. Whereas in feature extraction approach, augmented data is used to train target SVM classifiers. This will be further explained in the following sections. Figure 5 illustrates the flow chart diagram of how these approaches can be carried out.

### Evaluation of Tuned Classifiers

In this section, we discuss the setup of the tuned classifiers and the analysis of obtained results.

#### Tuned Classifier Setup

**Fig. 6:**
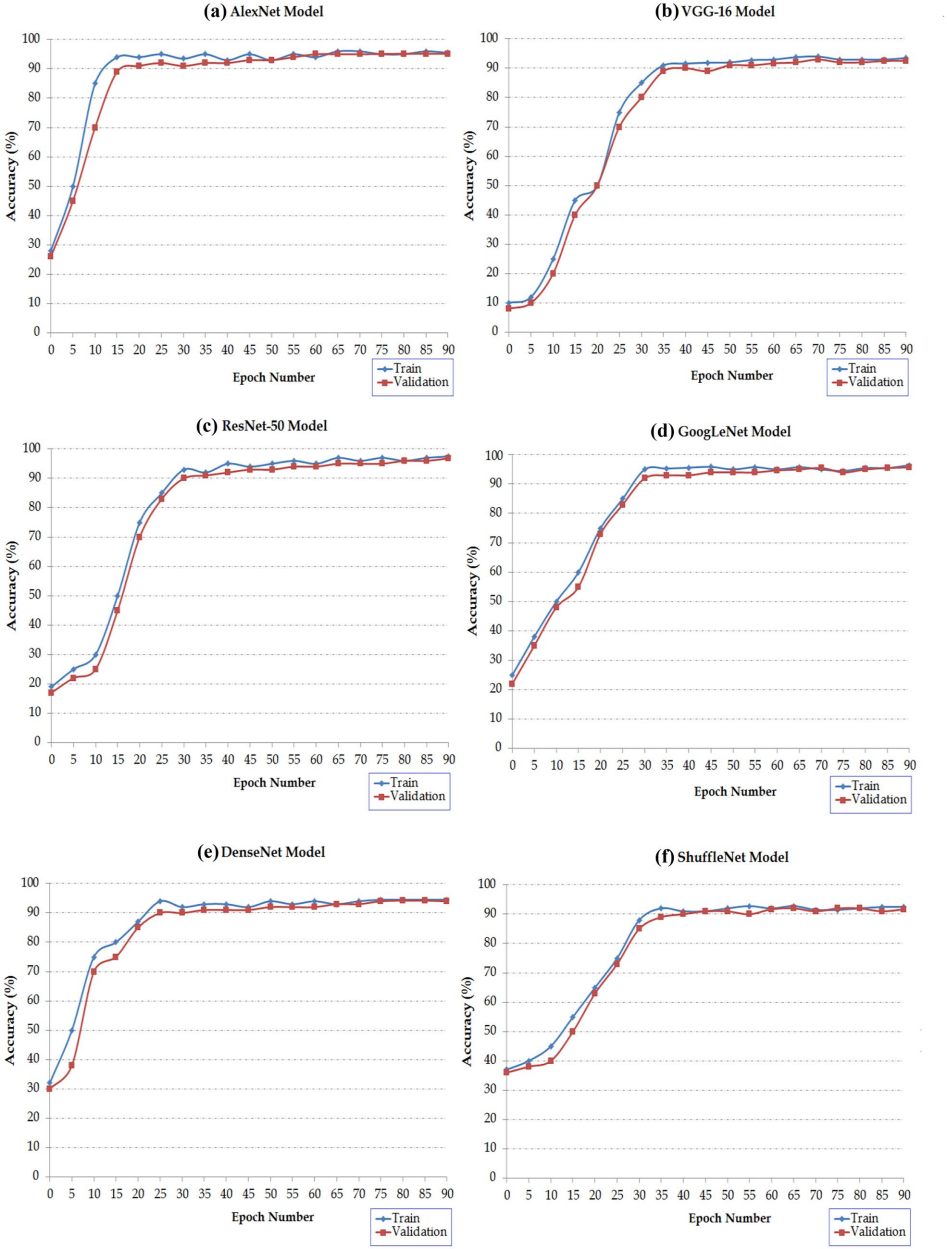
Visualisation of the training progress for different target networks

The tuned classifier setup is as follows:

- *For transfer learning*, to learn the new layers faster than the transferred layers, set the parameter values of the fully connected layer as learn rate factors (LRFs) to a large value, whereas initial learning rate (ILR) is to a small value. Therefore, weight learn rate factor, bias learn rate factor and initial learning rate values were set to 20, 20 and 0.0001, respectively. Then the target networks were retrained by augmented training data.
- *For network training*, target networks were run for 90 epochs. The training was done using stochastic gradient descent (SGD) with momentum set to 0.90 with a batch size of 128. The learning rate was initially set to 0.0001 as a starting point and was decremented after each update. The programme validated target networks every three iterations; 3 iterations per epoch were selected as the maximum number. The different models were trained and tested with the same training and testing sets for a fair comparison. Figure 6 shows the training process of the different fine-tuned classifiers.

#### Analysis of the Results Obtained

**Fig. 7:**
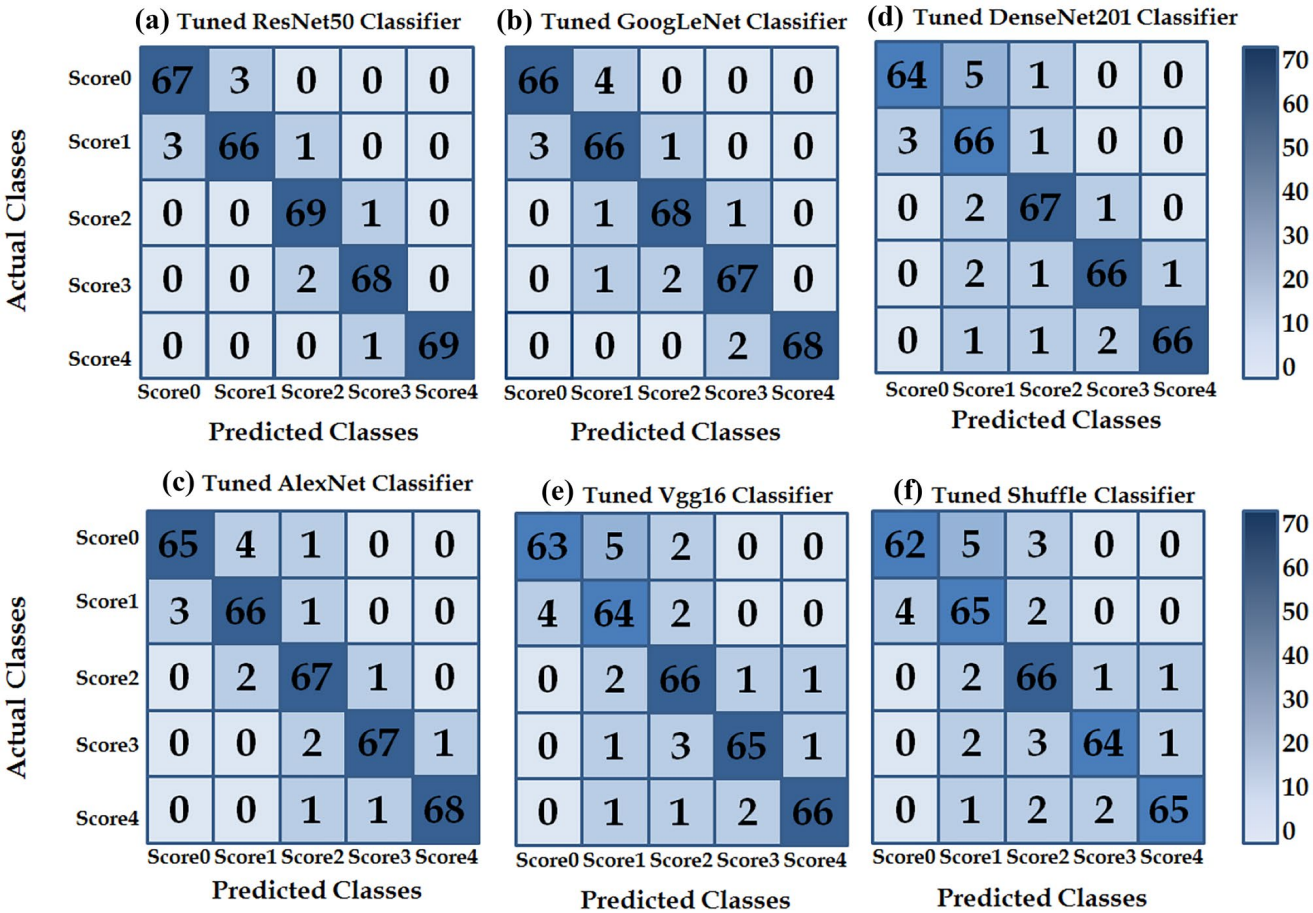
Confusion matrices for various fine-tuned CNN evaluations

The analysis of the results obtained is as follows:

- *Training progress analysis*: the analysis of the obtained results in Fig. 6 indicates that the fine-tuning models are rapidly gaining convergence during the training stage. Where the convergence approximately occurred in the first twenty epochs. This is also shown by Tajbakhsh et al. [31] indicating that the fine-tuning CNN approach gains convergence faster. From the 60th epoch, the accuracy rate was approximately steady for all target networks; i.e. the values of accuracy rates rose slowly. This is the main reason for ending the training at the 90th epoch.
- *Timing training analysis*: from the analysis of timing training of the six target networks, we found that AlexNet is the fastest; it took 45 min to train. For the same training data, VGG16, GoogLeNet, ResNet50, DenseNet and ShuffleNet took 90, 270, 120, 300 and 200 min, respectively.
- *Performance analysis*: 5 × 5 confusion matrices were used to represent the obtained prediction results of the cancerous pathological samples. Five scores represented the percentage of GLUT1 staining in tumour cells. Here, the *X*-axis represents the actual values, and the *Y*-axis represents the predicted values. These 5 × 5 confusion matrices are shown in Fig. 7. Statistical performance measurement results of different fine-tuned classifiers were summarised in Tables 1 and 2. Accuracy, recall, precision and F1-score metrics were computed by using Eqs. 5, 6, 7 and 4, respectively. As can be seen from Table 1, the higher successful classification was achieved by the tuned ResNet50 and tuned GoogLeNet with accuracy rates that were 96.86% and 95.17%, respectively. Followed by tuned AlexNet, DenseNet, VGG16 and Shuffle models with accuracy rates were 95.14%, 94.00%, 92.57% and 91.17%, respectively. In the same way, from Table 2, we find that the tuned ResNet-50 model has obtained the highest F1-score values than the other models.

**Table 1:** Accuracy metric for different tuned classifiers

*Fine-tuned model*	*Accuracy (%)*
Tuned AlexNet	95.14
Tuned VGG16	92.57
Tuned GoogLeNet	95.71
Tuned ResNet-50	96.86
Tuned DenseNet-201	94.00
Tuned ShuffleNet	91.71

**Table 2:** Standard metrics for various fine-tuned classifiers

*Proposed architecture*	*Metrics*	*IHC Score*				
		*Score0*	*Score1*	*Score2*	*Score3*	*Score4*
AlexNet	***P***_***i***_	0.93	0.94	0.96	0.96	0.97
	***R***_***i***_	0.96	0.92	0.93	0.97	0.99
	***F***_***1***_***score***	0.94	0.93	0.94	0.96	0.98
VGG16	***P***_***i***_	0.90	0.91	0.94	0.93	0.94
	***R***_***i***_	0.94	0.88	0.89	0.96	0.97
	***F***_***1***_***score***	0.92	0.89	0.91	0.94	0.95
GoogleNet	***P***_***i***_	0.94	0.94	0.97	0.96	0.97
	***R***_***i***_	0.96	0.92	0.96	0.96	1.00
	***F***_***1***_***score***	0.95	0.93	0.96	0.96	0.98
ResNet50	***P***_***i***_	0.96	0.94	0.99	0.97	0. 99
	***R***_***i***_	0.96	0.96	0.96	0.97	1.00
	***F***_***1***_***score***	0.96	0.95	0.97	0.97	0.99
DenseNet	***P***_***i***_	0.91	0.94	0.96	0.94	0.94
	***R***_***i***_	0.96	0.87	0.94	0.96	0.99
	***F***_***1***_***score***	0.93	0.90	0.95	0.95	0.96
ShuffleNet	***P***_***i***_	0.89	0.93	0.94	0.91	0.93
	***R***_***i***_	0.94	0.87	0.87	0.96	0.97
	***F***_***1***_***score***	0.91	0.90	0.90	0.93	0.95

### Evaluation of the SVM Classifiers

In this methodology, the experiment was carried out by using the target SVM classifiers that have been trained on the extracted deep feature vectors from the activation layer of the six proposed extractors. The extracted deep feature vectors are illustrated in Table 3. The flow diagram of the steps involved in the experiment is illustrated in Fig. 5. As in the previous section, we discuss the setup of the SVM classifiers and then the analysis of obtained results.

**Table 3:** Feature vectors for different activation layers

*Feature Vector*	*Activation Layer*	*Feature*
*Feat-Alex*	“fc7”	1 × 1 × 4096
*Feat-Vgg16*	“fc7”	1 × 1 × 4096
*Feat-GoogLe*	“pool5-7 × 7-s_1_”	1 × 1 × 1024
*Feat-Res50*	“avg-pool”	1 × 1 × 2048
*Feat-Dense*	“avg-pool”	1 × 1 × 1920
*Feat-Suffle*	“node-200”	1 × 1 × 544

#### SVM Classifier Setup

The target SVM classifiers were set up by combining them with an error-correcting output codes (ECOC) function as described in [41]. ECOC function is commonly used for modelling a multi-class classification problem. It divides a K class problem into $\frac{K\left(K-1\right)}{2}$ binary learners, then assigns a one-versus-one coding design to determine the classes for such binary learning [41]. Setting SVM classifiers with the ECOC framework could improve classification accuracy, even compared to other multi-class models.

#### Analysis of the SVM Classifiers’ Performance

**Fig. 8:**
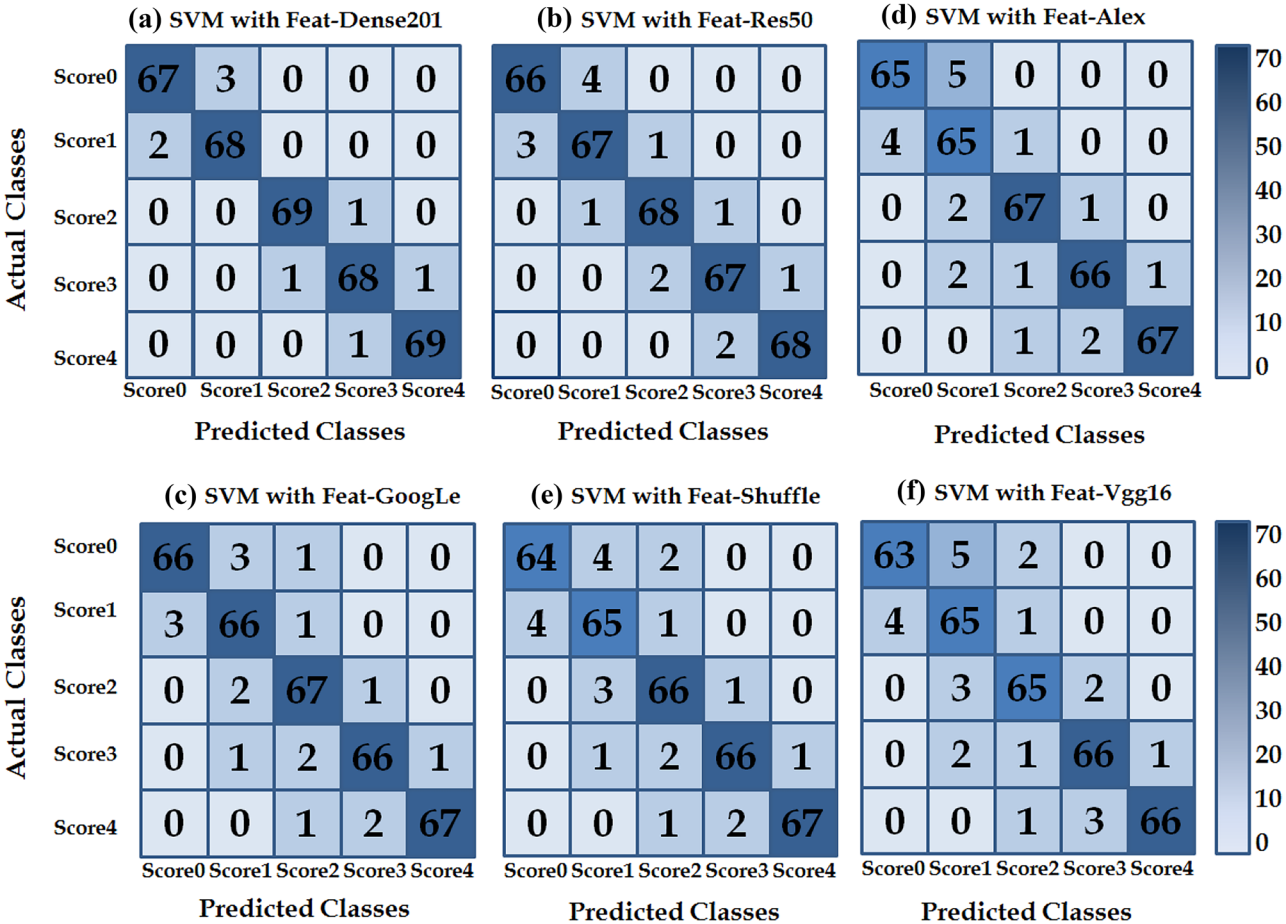
Confusion matrices for SVM classifiers trained on different feature vectors

- By the analysis of timing training, we recorded that classifier training time on *Feat-Alex*, *Feat-Vgg16*, *Feat-GoogLe*, *Feat-Res50*, *Feat-Dense* and *Feat-Suffle* took 29, 35, 49, 44, 58 and 32 min, respectively.
- The obtained results of the experiment were represented in the 5 × 5 confusion matrices as shown in Fig. 8. Statistical performance measurement results of SVM classifiers were reported in Tables 4 and 5. The performance results were computed by using Eqs. 5, 6, 7 and 4. It can be noted that the trained SVM classifier on the *Feat-Dense* vector has obtained the highest value than the other vectors in terms of classification accuracy (97.43%), recall, precision and F1-score values.

**Table 4:** Accuracy metrics for SVM Classifiers

*Trained SVM classifier with*	*Accuracy (%)*
*Feat-Alex*	93.70
*Feat-Vgg16*	92.86
*Feat-GoogLe*	94.85
*Feat-Res50*	96.00
*Feat-Dense*	97.43
*Feat-Shuffle*	94.28

**Table 5:** Standard metrics for SVM classifiers

*Feature vector*	*Metrics*	IHC Score				
		*Score0*	*Score1*	*Score2*	*Score3*	*Score4*
Feat-Alex	***P***_***i***_	0.93	0.93	0.96	0.94	0.96
	***R***_***i***_	0.94	0.88	0.96	0.96	0.99
	***F***_***1***_***score***	0.93	0.90	0.96	0.95	0.97
Feat-VGG16	***P***_***i***_	0.90	0.93	0.93	0.94	0.94
	***R***_***i***_	0.94	0.87	0.93	0.93	0.99
	***F***_***1***_***score***	0.92	0.90	0.93	0.93	0.96
Feat-GoogLe	***P***_***i***_	0.94	0.94	0.96	0.94	0.96
	***R***_***i***_	0.96	0.92	0.93	0.96	0.99
	***F***_***1***_***score***	0.95	0.93	0.94	0.95	0.97
Feat-Res50	***P***_***i***_	0.94	0.96	0.97	0.96	0.97
	***R***_***i***_	0.96	0.93	0.96	0.96	0.99
	***F***_***1***_***score***	0.95	0.94	0.96	0.96	0.98
Feat-Dense	***P***_***i***_	0.96	0.97	0.99	0.97	0.99
	***R***_***i***_	0.97	0.96	0.99	0.97	0.99
	***F***_***1***_***score***	0.96	0.96	0.99	0.97	0.99
Feat-Shuffle	***P***_***i***_	0.91	0.93	0.94	0.94	0.96
	***R***_***i***_	0.94	0.89	0.92	0.96	0.99
	***F***_***1***_***score***	0.92	0.91	0.93	0.95	0.97

### Evaluation of the Proposed Feature Selection Approach

**Fig. 9:**
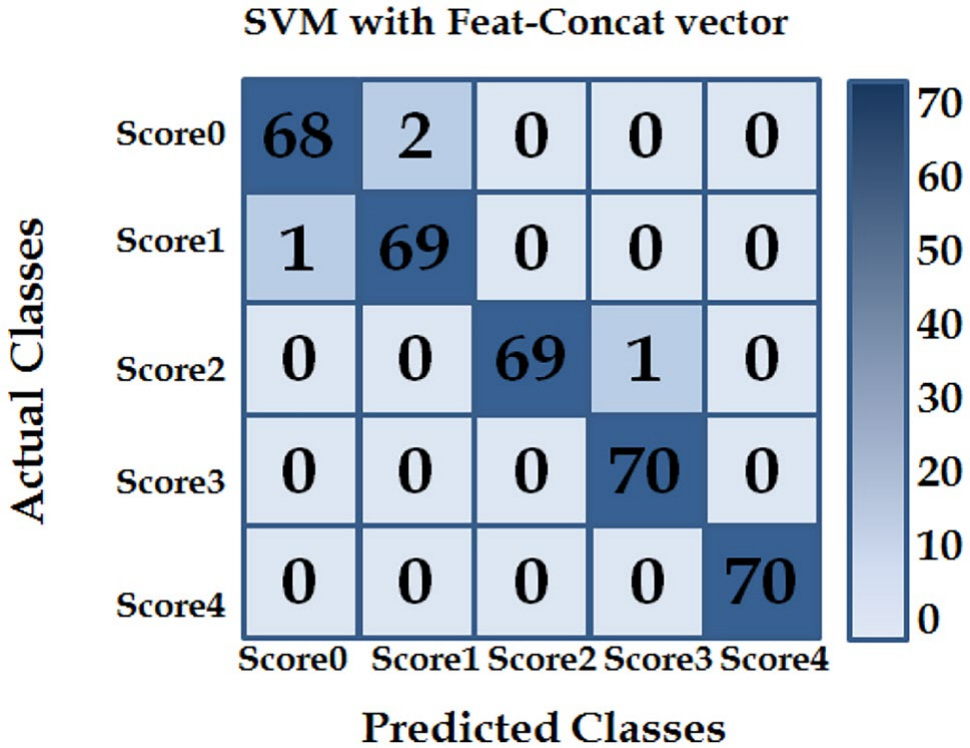
Confusion matrix for SVM Classifier with *Feat-Concat* vector

In the previous experiment, Feat-Dense201, Feat-Res50 and Feat-GoogLe vectors won the three best accuracies, which is why they were chosen in this experiment. Several studies [42–44] have employed the feature selection approach to improve classification accuracy and reduce over-fitting and training time. The goal of this experiment was to determine the features that yield better performance than the others. We concatenate these superior deep features and select the best features for representing our application to improve the performance. We employed a sparse support vector machine (SSVM) classifier to select the best deep features into a Feat-Concat vector. Feat-Concat vector has been utilised for training other SVM with ECOC classifier. The performance metric of the SVM classifier that trained on the Feat-Concat vector is given in Table 6, and Fig. 9 illustrates a 5 × 5 confusion matrix. It can be seen from Table 6 that the SVM classifier achieved the highest accuracy rate of 98.86%. It performed the classification with better accuracy than the other classifiers. The SSVM [41] solves the optimisation problem by minimising the following equation:

8 $$SSVM=\frac{1}{n}\sum_{i=1}^{n}\left[1-{{\varvec{y}}}_{i}\left(b+{\varvec{z}}h({{\varvec{x}}}_{i})\right)\right]+\lambda \left|{\varvec{z}}\right|,$$

where *n* is the number of input images, ${x}_{i}=\left({x}_{i,1}, {x}_{i,2}, {x}_{i,3},\dots ,{x}_{n,d}\right)$ is a vector of the *i*th feature, *d* is the number of feature, and ***y***_i_ is the class label and belonged to {+ 1, − 1}, for *i* = 1,…, *n*, where *y*_*i*_ = + 1 indicates the *i*th sample is in class 1 (e.g. has cancer) and *y*_*i*_ = − 1 indicates the *i*th sample is in class 2 (e.g. does not have cancer). ***z*** is a hyper-plane parameter, $\left[1-{{\varvec{y}}}_{i}\left(b+{\varvec{z}}h({{\varvec{x}}}_{i})\right)\right]$ is the convex hinge loss function, the scalar *b* is denoted as the bias, $\lambda \left|{\varvec{z}}\right|$ is the L_1_-norm, and λ > 0 is the tuning parameter controlling the trade-off between minimising the hyper-plane coefficients and the classification error.

**Table 6:** Performance metric for SVM classifier with *Feat-Concat* vector

*Metrics*	*IHC score*				
	*Score-0*	*Score-1*	*Score-2*	*Score-3*	*Score-4*
Pi	0.97	0.99	0.99	1.00	1.00
Ri	0.99	0.97	1.00	0.99	1.00
F1score	0.98	0.98	0.99	0.99	1.00

## Discussion and Conclusion

In the discussion part, we will provide an analytical comparison among the methodologies used in the three experiments as follows:

- Fine-tuning ResNet-50 architecture is more suitable for our application than the other proposed architectures.
- The transferable deep features of DenseNet architecture are learned more than the deep features of other proposed architectures. Therefore the performance of the SVM classifier that is trained on the Feat-Dense vector has outperformed the other vectors on our dataset images.
- By comparing the time taken to train classifiers, we find that extracting and training SVM classifiers are much less than fine-tuning the CNN model as a classifier. Therefore, off-the-shelf feature extraction methodology is better than fine-tuning CNN methodology in terms of performance, training time and resources required for training.
- Although several studies [14, 16, 28, 35] have compared deep transfer learning methodologies in the histopathological field, there is no consensus about whether one is better than the other. However, this work showed that using off-the-shelf features methodology yielded performances slightly superior to using fine-tuning methodology but with the advantage of not having to re-train the network.
- The feature selection approach is proposed in this work to improve the performance of the SVM classifier for scoring tumour hypoxia markers as it yields optimal performance. Therefore, our contribution is to introduce the *Feat-Concat* vector to train the SVM predictor.
- Our study does have some limitations. Firstly, the proposed framework was implemented during COVID-19, so the resources were restricted. Secondly, the used dataset images were prepared in the same laboratory and under the same conditions (i.e. dataset images acquired with the same digital scanners and staining techniques). Finally, choosing the appropriate patches was one of the difficulties that we faced in carrying out the experiments.

In conclusion, this paper proposed a deep learning-based framework to automatically assess the GLUT1 scoring as a biomarker of tumour hypoxia in IHC images. It helps to avoid inter-observer disagreement between pathologists and improves diagnostic performance. Three experiments were carried out using two common deep transfer learning methodologies with six various CNN architectures for classifying tumour hypoxia markers scores. From the results obtained in our experiments, it is observed that deep transfer learning approaches can significantly improve classification accuracy. Therefore, it is the best strategy in case of scarcity of dataset images, as is the case with histopathological images. This may be considered an initial step towards developing a reliable computer-assisted diagnosis tool for GLUT-1 scoring of digitised colorectal cancer histology slides. The future indications include the extension of our dataset and the inclusion of IHC images of different cancers from various laboratories. Also, state-of-the-art pre-trained models need to be included in future work. Finally, it will be targeted to apply deep transfer learning to develop a comprehensive computer-assisted diagnosis tool for GLUT-1 scoring of diverse cancerous tissue slides.

## Data Availability

The image dataset can be made available.
